# Modelling and Thermographic Measurements of LED Optical Power

**DOI:** 10.3390/s24051471

**Published:** 2024-02-24

**Authors:** Maria Strąkowska, Sebastian Urbaś, Mariusz Felczak, Błażej Torzyk, Iyad S. M. Shatarah, Rafał Kasikowski, Przemysław Tabaka, Bogusław Więcek

**Affiliations:** 1 Institute of Electronics, Lodz University of Technology, 90-924 Lodz, Poland; sebastian.urbas@dokt.p.lodz.pl (S.U.); mariusz.felczak@p.lodz.pl (M.F.); iyad.shatarah@p.lodz.pl (I.S.M.S.); rafal.kasikowski@p.lodz.pl (R.K.); boguslaw.wiecek@p.lodz.pl (B.W.); 2 Institute of Electrical Power Engineering, Lodz University of Technology, 90-924 Lodz, Poland; przemyslaw.tabaka@p.lodz.pl

**Keywords:** IR thermography, LED, optical power, thermal modelling

## Abstract

This paper presents a simple engineering method for evaluating the optical power emitted by light-emitting diodes (LEDs) using infrared thermography. The method is based on the simultaneous measurement of the electrical power and temperature of an LED and a heat source (resistor) that are enclosed in the same plastic packaging under the same cooling conditions. This ensures the calculation of the optical power emitted by the LED regardless of the value of the heat transfer coefficient. The obtained result was confirmed by comparing it with the standard direct measurement method using an integrated sphere. The values of the estimated optical power using the proposed method and the integrated sphere equipped with a spectrometer were consistent with each other. The tested LED exhibited a high optical energy efficiency, reaching approximately η ≈ 30%. In addition, an uncertainty analysis of the obtained results was performed. Compact modelling based on a thermal resistor network (R_th_) and a 3D-FEM analysis were performed to confirm the experimental results.

## 1. Introduction

The optical power of light sources is usually measured with Ulbricht integrating spheres [1], which are equipped with calibrated and certified spectrometers for precise spectral measurements. This means that optical power measurements can only be performed in laboratories equipped with advanced measurement systems. In the general case, this ensures the high accuracy of optical power measurements. There is another approach to such measurements using goniometers, which is more difficult to apply in practice. It requires a dark room, and the entire measurement is performed step by step for each emission direction. It is characterized by a long measurement time and is obviously not suitable for use in the case of diodes operating in a lighting system in the field.

In many practical cases, there is a need to measure the optical power of LEDs using simple technical methods. Typically, LEDs emit light in one or more narrow bands. In addition, due to production tolerances, manufacturers indicate the light intensity or luminous flux with a large scatter, which causes difficulties in selecting the appropriate element for new solutions where an exact value of radiant power is required.

Nowadays, highly efficient LEDs are available, in which a significant part of the electrical power is converted into radiation [2,3,4,5]. On the other hand, the high optical power emitted involves a large amount of thermal power dissipated, which increases the temperature of the device. Knowing the radiant power helps to develop effective heat management for LED light sources. The radiation characteristics of LEDs are expressed in terms of either photometric or radiometric parameters. The photometric parameters of light sources are calculated and measured in relation to green color radiation at a wavelength of λ = 555 nm. Typically, manufacturers provide the photometric parameters, while for energy analyses, radiometric data are needed [6].

Measuring the emitted infrared radiation and the electrical power consumption allows one to obtain the optical power and efficiency of an LED.

Thermography, as a noninvasive, cost-effective, and fast method, could be applied in many fields of engineering practice, including electronics [7,8]. In electronics, it is often used for estimating the heat loss of components and entire systems. In many cases, it can replace much more expensive and sophisticated measurement equipment. The Joule heating of an LED can be compared with the Joule heating of a resistor, both being encapsulated in identical housings. Measuring the emitted infrared radiation and the electrical power consumption allows one to obtain the optical power and efficiency of an LED. The results of the thermographic method and the radiometric measurement of the luminous flux using an integrating sphere and calibrated spectrometer are presented in this paper. This paper considers both thermal and radiation problems and hence both research domains are taken into account. Heat transfer analysis can support the investigations of optical phenomena. In the literature, there are numerous thermal analyses of LED structures based on different modelling approaches, such as 3D-FEM (finite element method), analytical conjugate analyses, and compact simplified models [4,9,10,11,12,13]. In this research, a heat transfer analysis using compact 3D-FEM modelling was performed to validate the obtained experimental results.

## 2. Materials and Methods

### 2.1. Thermal Modelling

**Figure 1:**
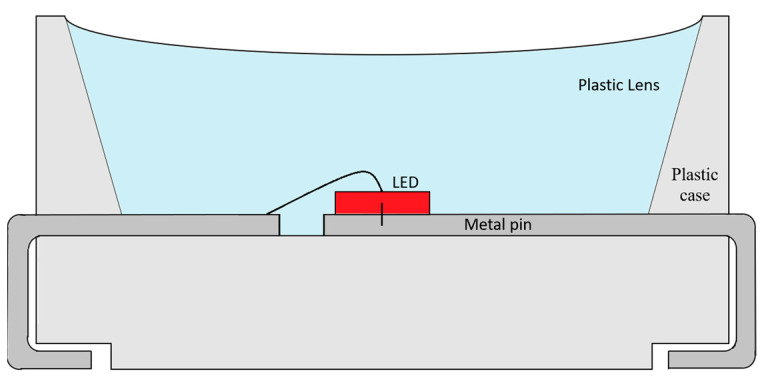
Cross-section of a high-efficiency LED.

High-efficiency LEDs were sealed in a variety of plastic packages. An example cross-section of a 5 × 5 mm^2^ package used in this research is shown in Figure 1. It is an RGB module with semiconductor diodes placed in a row in the middle of the substrate. Metal connectors/electrodes play a very important role in heat dissipation. Above the semiconductors is a semi-transparent diffusive material that acts as an optical lens and provides an emission angle of 110°. The rated current is 20 mA. For the red diode, the forward voltage is about 2.0 V and the typical luminous intensity is 1090 mcd. The central wavelength is 620 nm and the full width at half maximum (FWHM) wavelength is about 20 nm.

In this research, an R-LED was considered. This light source was attached to the substrate closer to one side of the housing. This research began with heat transfer modelling to estimate the temperature rise above the ambient temperature. Two models were developed: a simple, compact one for rapidly estimating the steady-state LED temperature using Matlab R2023b software, and a 3D model implemented in the ANSYS R19.1 environment using the FEM to solve heat transfer differential equations. For the external walls of the model, convective boundary conditions were set. The model assumptions were comparable with R_th_ network compact thermal modelling. In the compact R_th_ network model, the power cables were modelled as infinite wires, while in the FEM, the boundary conditions were modified to consider heat dissipation by the powering connections. In this way, heat transfer through the wires was simulated by increasing the values of the heat transfer coefficients. The simulation was carried out for power of *P* = 27.72 mW dissipated in the diode, corresponding to the thermal power obtained in measurements.

#### 2.1.1. R_th_ Network—Compact Thermal LED Model

**Figure 2:**
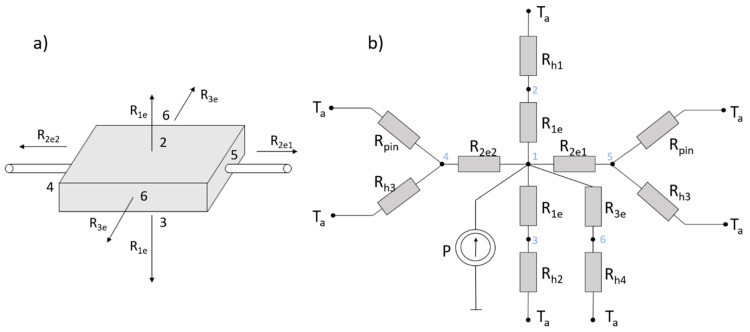
Scheme of thermal model of a diode (**a**) and R_th_-network compact thermal model of an LED (**b**).

A simple thermal compact LED model consisting of a network of thermal resistances *R_th_* is shown in Figure 2. The heat source is inside the plastic housing. A part of the generated thermal power *P_TD_* is dissipated to the environment in all directions: vertically to the top and bottom surfaces, represented by the thermal resistance *R*_1*e*_, and horizontally to all four side surfaces, represented by *R*_2*e*1_, *R*_2*e*2_, and *R*_3*e*_. A significant part of the dissipated power is transferred through the electrical connectors soldered to the LED pins (*R_pin_*). Finally, all power is transferred to the environment by free convection, modelled using the heat transfer coefficients represented by thermal resistances *R_h_*_1_, *R_h_*_2_, *R_h_*_3_, and *R_h_*_4_.

The compact thermal model of an LED based on R_th_ network consists of six main branches corresponding to six side sides of the LED housing, as shown in Figure 2. The electrical power is divided into optical and thermal parts. The dissipated thermal power *P_TD_* is assumed to be transferred through thermal resistances representing heat conduction and free convection. The material parameters and dimensions of each part of the diode are given in Table 1. Convective heat transfer coefficients were estimated using the optimization method in order to match the model results to the results obtained in the measurements [4,8].

The resistance *R*_1*e*_ associated with heat conduction to the top and bottom surfaces of the diode takes the form (1):

(1) $$R_{1e}=\frac{d_{1}}{k_{epoxy}S_{1e}}$$

The resistance *R_h_*_1_ corresponding to convection cooling is expressed as (2):

(2) $$R_{h1}=\frac{1}{h_{1}S_{d}}$$

**Figure 3:**
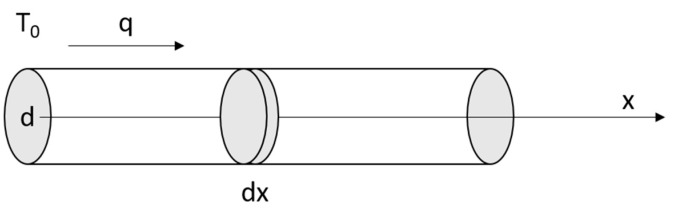
Connection wire model.

Temperature measurements with the IR camera take place on the upper surface of the diode (node 2). On both sides (left—node 4, right—node 5), there are connecting wires that transfer a large part of the heat to the environment—*R_pin_*. The value of *R_pin_* is calculated assuming an infinite length of the wire in Figure 3.

The one-dimensional thermal model of the wire is represented by Equations (3)–(5).

(3) $$-dq*\pi r^{2}=2\pi r*dx*hT$$

(4) $$q=-k\frac{dT}{dx}$$

(5) $$\frac{d^{2}T}{dx^{2}}-\frac{2h}{rk}T=0$$

The temperature *T* in all equations is the temperature difference between the thermal object and the environment. In other words, we assumed that the ambient temperature is equal to 0. In practice, this means that in order to find the real object temperature when modelling, the actual ambient temperature must be added to the temperature obtained by the model.

The general solution of (5) takes the form:

(6) $$T=T_{0}e^{-\frac{x}{L}}$$

where the diffusion length *L* is expressed as:

(7) $$L=\sqrt{\frac{rk}{2h}}$$

The boundary condition for *x* = 0 allows us to obtain a special solution of (5).

(8) $${\left.\frac{dT}{dx}\right|}_{x=0}=-\frac{T_{0}}{L}$$

(9) $$q=-\frac{kT_{0}}{L}=-\frac{P}{\pi r^{2}}$$

Finally, the thermal resistance of the wire *R_pin_* can be presented as (10).

(10) $$R_{pin}=\frac{T_{0}}{P}=\frac{L}{k\pi r^{2}}=\frac{1}{\pi r}\sqrt{\frac{1}{2hkr}}$$

On the left side of the diode, there is a thin wire leading to the diode’s left electrical contact—Figure 1. The thermal resistance here, *R*_2*e*2_, is slightly different to the resistance on the right side, *R*_2*e*1_, where the diode lies directly on the thermal pad. The equations of thermal resistances for these branches are given by (11)–(13):

(11) $$R_{2e2}=\frac{d_{2}}{k_{Cu}*S_{2e}}$$

(12) $$R_{2e1}=\frac{d_{Cu}}{k_{Cu}*S_{2e}}$$

(13) $$R_{h3}=\frac{1}{h_{3}*S_{3e}}$$

The two other branches (back and forward) are the same, so they are analyzed together as node 6. The corresponding resistances are given by (14) and (15).

(14) $$R_{3e}=\frac{d_{2}}{k_{epoxy}*{2*S}_{2e}}$$

(15) $$R_{h4}=\frac{1}{h_{3}\cdot 2\cdot S_{2e}}$$

#### 2.1.2. Three-Dimensional FEM Model

A 3D FEM model was developed in the ANSYS environment (version R19.1). For geometry preparation, Space Claim software was used (part of the ANSYS R19.1 software package). The dimensions and material properties correspond to the R_th_ network compact thermal model as well as the real diode and package sizes.

In this case, steady-state simulation was performed. For this purpose, Workbench software (part of the ANSYS R19.1 software package) was used. The simulation type was Steady-State Thermal.

### 2.2. Thermographic Method of LED Optical Power Evaluation

**Figure 4:**
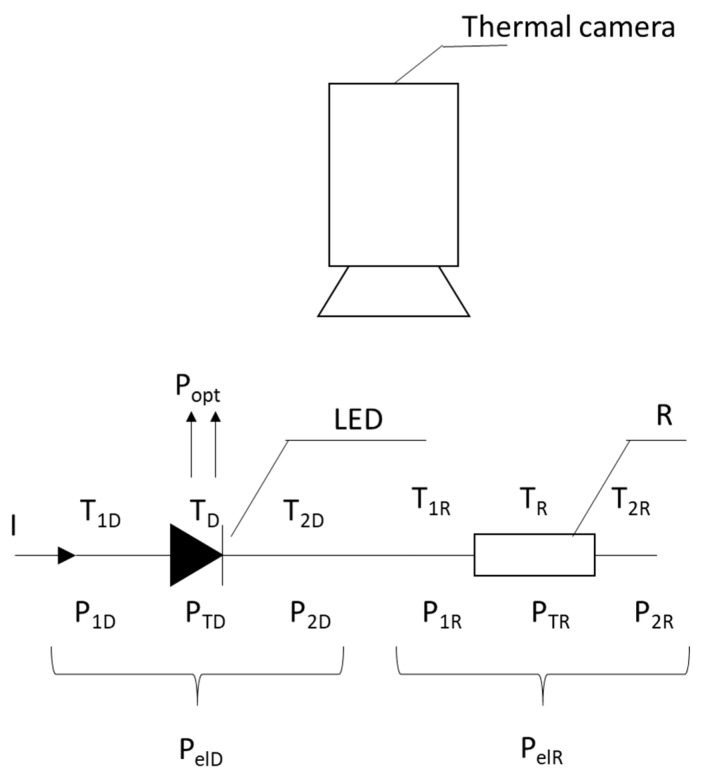
The concept of thermovision measurements of the optical power of LEDs.

The proposed method consists of the simultaneous measurement of the temperature of the tested LED, *T_D_*, the heating element (resistor—R), *T_R_*, and the power cables on both sides of the elements: *T*_1*D*_, *T*_2*D*_, *T*_1*R*_, and *T*_2*R*_. All measurements were performed using a thermal imaging camera, as shown in Figure 4. The emissivity values of the measured elements were estimated at the value ε = 0.92.

**Figure 5:**
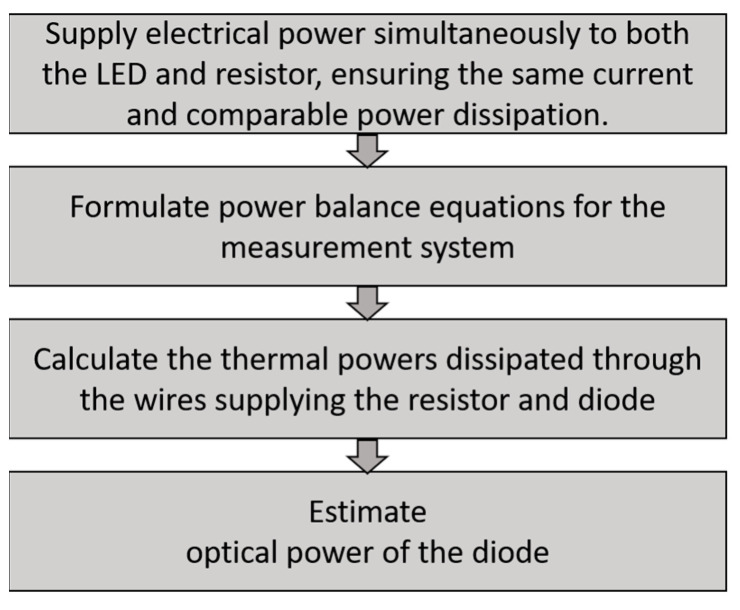
Block diagram of the proposed measurement method for LED optical power.

The flowchart illustrating the method and experiment is shown in Figure 5.

Electrical power, *P_elD_* and *P_elR_*, is supplied to both elements connected in series simultaneously. This allows us to obtain the same current and comparable power dissipated in them. The key issue of the proposed method is to ensure equal convection cooling conditions of both elements: the LED diode and resistor R. This can be achieved by using the same shape and geometric dimensions for both elements. This will ensure equal conditions for heat dissipation from them to the environment. In addition, there should be equal and stable thermal cooling conditions around the LED and the resistor R during the measurement. This makes it possible to obtain identical values of the averaged convective and radiation heat transfer coefficient *h* for both elements.

As a result, power balance equations can be obtained for the measurement system shown in Figure 4:

(16) $$P_{elD}=P_{TD}+P_{opt}=hT_{D}+P_{12D}+P_{opt}$$

(17) $$P_{elR}=P_{TR}=hT_{R}+P_{12R}$$

where *P_elR_* and *P_elD_*—the electrical power supply of the resistor and the diode, respectively, *P_TR_* and *P_TD_*—thermal powers dissipated to the environment by the resistor and the diode, respectively, *P*_12*R*_ and *P*_12*D*_—thermal powers dissipated to the environment through the supply wires of the resistor and the diode, respectively, *P_opt_*—radiant power of the diode, *T_R_* and *T_D_*—temperature differences between the resistor and diode and the ambient temperature, respectively, and *h*—heat transfer coefficient.

**Figure 6:**
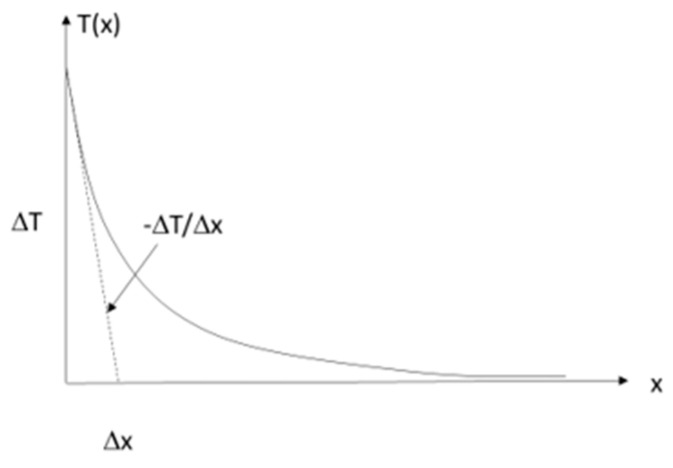
Determination of the thermal power dissipated to the environment through the wires supplying the resistor and the diode.

The thermal power dissipated to the environment through the resistor and diode connecting wires is calculated by determining the temperature gradient along the power cables on both sides of each element, *P*_1*D*(*R*)_ and *P*_2*D*(*R*)_, with the knowledge of the cable thermal conductivity coefficient *k* and its diameter *2r*—Figure 6.

The thermal power transferred to the environment through the wires supplying the resistor and the diode can be determined by:

(18) $$P_{12D(R)}=P_{1D(R)}+P_{2D(R)}=-k\frac{∆T_{1D(R)}}{∆x}\pi r^{2}-k\frac{∆T_{2D(R)}}{∆x}\pi r^{2}$$

where *T*_1*D*_ and *T*_2*D*_—temperature of the diode supply cable on side 1 and 2, respectively, and *T*_1*R*_ and *T*_2*R*_—temperature of the cable supplying the resistor on side 1 and 2, respectively.

From Equations (16) and (17), the optical power *P_opt_* of the diode is finally expressed as:

(19) $$P_{opt}=P_{elD}-P_{12D}-\frac{T_{D}}{T_{R}}(P_{elR}-P_{12R})$$

### 2.3. Measurement Setup

**Figure 7:**
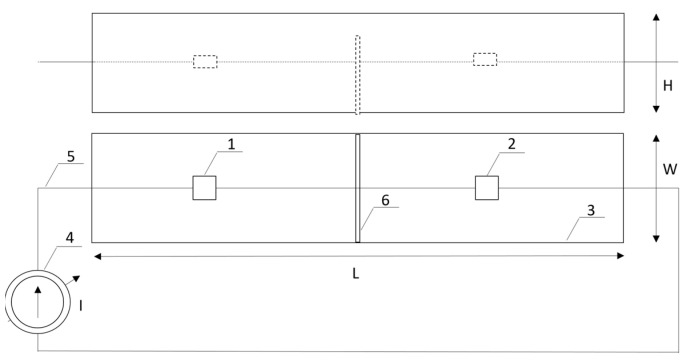
Experimental setup and the tray with the measured elements: resistor R (1) and an LED (2).

A diagram of the measuring system is presented in Figure 7. The system consists of a resistor (1) and an LED (2). Both elements, the LED and resistor R, are connected in series and supplied from the current source (4) with an adjustable current in the range of 10–30 mA. Both measuring elements (LED and R) were placed in a trough (3) with dimensions of L = 25 cm, W = 10 cm, and H = 5 cm. In order to ensure the lack of thermal impact of both heat sources, the distance between them should not be less than 15 cm. A support (6) placed in the middle of the trough separates and insulates both elements. The tested elements were suspended in the trough by means of power supply wires at a height of approx. 1/2 of the height H from the bottom of the trough to ensure stable thermal conditions during the measurement. The trough was closed except for the upper part, over which the thermal imaging camera was placed. The trough with the support was made via 3D printing. Similarly, using the same method, a thermal element (1) was made, in which a heat source in the form of a small resistor with dimensions similar to the dimensions of the semiconductor structure of the LED diode was embedded.

**Figure 8:**
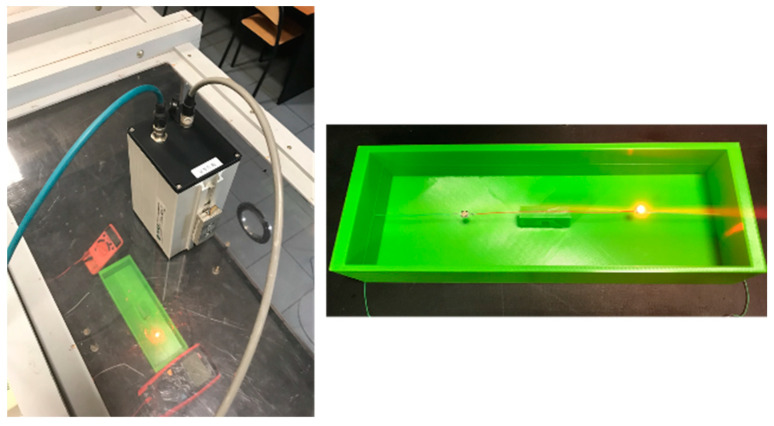
Scheme of the measurement system.

A special measurement stand equipped with an IR microbolometer camera, a precise, adjustable current source, and current/voltage meters was prepared. Measurements were performed under laboratory conditions inside a closed chamber where the free convection is stable and homogenous. Airflow was reduced to a minimum throughout the experiment. The IR camera was places on top of the measurement section and it could monitor the temperature of the objects through a dedicated speculum made for an IR camera in the measurement section of the stand—Figure 8.

In order to prepare a non-radiant heat source, an SM-0603 surface-mounted resistor was inserted into the LED housing using 3D printing.

## 3. Results and Discussion

**Figure 9:**
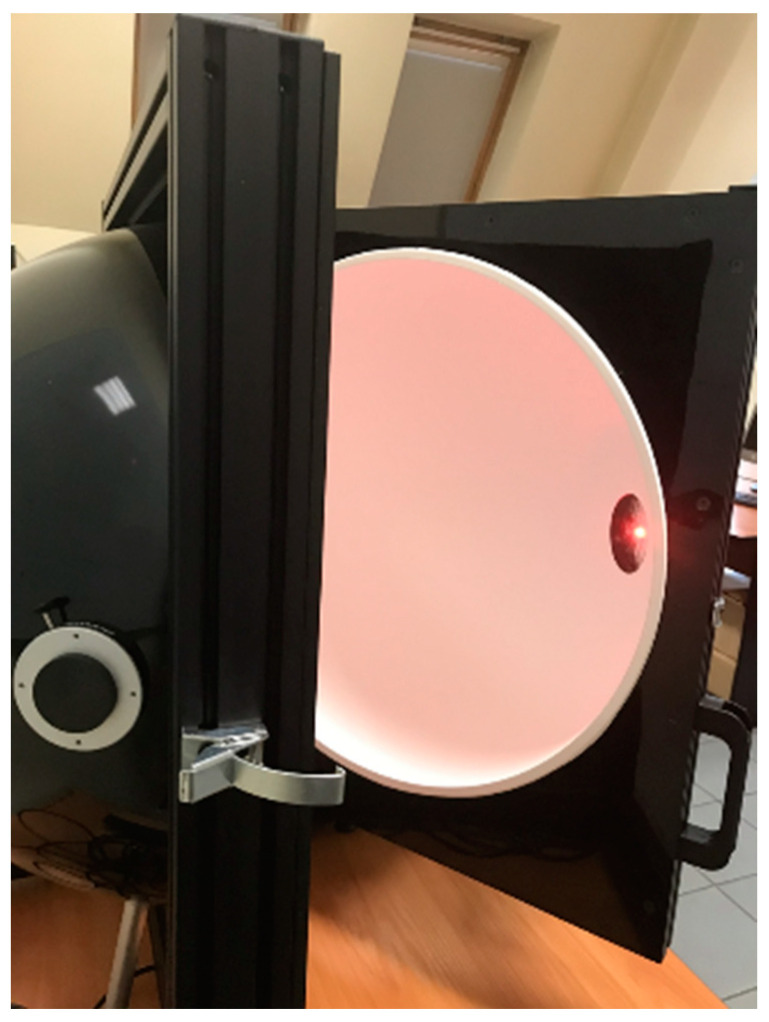
Integrating sphere with a mounted LED.

In order to compare the obtained results, the optical power emitted by the LED was measured directly using an integrating sphere—Figure 9. Using an integrated sphere is the most reliable and standard method for measuring radiant power, commonly used by light source producers, including LED manufacturers. This direct measurement is the standard method in this field. The used integrated sphere was calibrated in order to obtain proper values of emitted radiant power. A sphere with a diameter of 500 mm was equipped with a spectrometer, enabling the measurement of luminous flux and radiant power in the spectral range of λ ∊ (340–1700) nm.

In order to power the analyzed elements, a precise laboratory power supply was applied. For measuring the voltage and current of the diode and the resistor, two digital multimeters were used: one as a voltmeter and the other as an ampere meter. A *DIAS 640Lc* thermal imaging camera with *Pyrosoft* Compact software ver. 3.4.1.1 was used for the tests. This thermal imaging camera allows for precise non-contact temperature measurements from −20 °C to 500 °C. The IR camera has an uncooled microbolometer array with 640 × 480 pixels operating in the spectral range of 8–14 µm. The measurement frequency of this IR camera was 50 frames per second and the *NETD* (*Noise Equivalent Temperature Difference*) was less than 0.08 K.

### 3.1. Modelling Results

The result of the simulation of the R_th_ network thermal model presented in Section 2 gives the values of temperatures in all nodes (Table 2).

As can be seen, the temperature inside the diode housing is almost uniform. The temperature rise in node 2 corresponds to the temperature value on the upper surface of the diode, which was measured during the experiment and estimated to be 6.93 °C for a thermal power equal to *P_TD_* = 27.72 mW.

**Figure 10:**
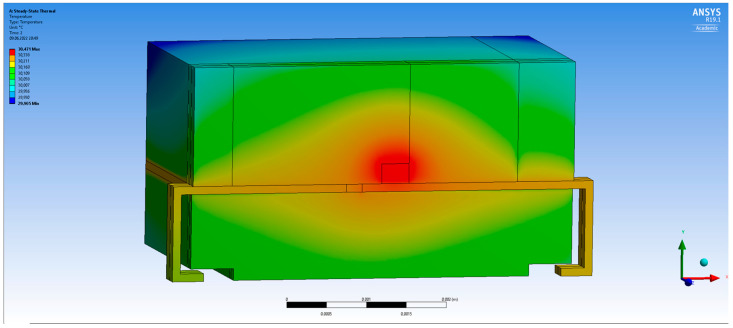
Temperature distribution inside the LED enclosure in a steady state.

An example of the simulation results obtained using the 3D-FEM model is shown in Figure 10.

The initial temperature as well as the boundary air temperature was set to 25 °C. The results of the modelling, presented as the values of the temperature at the specified measurement points, are shown in Table 3.

The geometry is not very complex; thus, the 3D model could be used without incurring a very long computation time. It took about 10 h using a desktop computer with an AMD EPYC 7301 16-Core Processor, 2.20 GHz, and 128 GB of RAM, which is not much for a 3D model.

Simulations of developed models were performed based on the shape and the structure of the measured diode and including the conditions of the measurement setup. In the FEM analysis in ANSYS, the mesh was hexahedral and almost regular in the whole simulated structure. Its side dimensions were equal to about 5 × 10^−5^ m, which balances the computation time and results accuracy.

The simulation was carried out for a thermal power equal to 27.72 mW. The temperature difference between the diode and the environment was 6.93 °C in the measurement, the same as the R_th_ model (node 2), and 6.95 °C in the 3D FEM analysis. Moreover, the temperature distributions in the corresponding nodes of the R_th_ compact model and surfaces from the 3D FEM are comparable. Both thermal models gave similar results—Table 2 and Table 3. The difference between the results could be reduced by using a denser mesh and a more accurate geometry.

### 3.2. Optical Power Evaluation

**Figure 11:**
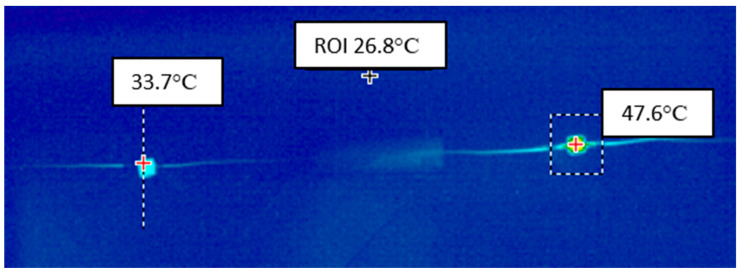
Thermogram of a series connection of the diode (on the left) and the resistor (on the right).

Optical power measurements were separated into six experimental sessions that lasted a few hours in one day. Each session consisted of six measurements to average the results. The maximum temperature of both the LED and the resistor was taken for the calculations. The average temperature could also be considered, but the maximum temperature was selected for further calculations due to the difficulties in precisely determining the LED area for averaging. The experiments were carried out with RGB LEDs in 5 × 5 mm housings and a non-radiating resistive heat source with resistance *R* = 220 Ω. During this experiment, the thermal resistance of the LED package inside the measuring stand was estimated at approximately *R_th_* ≈ 255 K/W. Exemplary results of temperature measurements using the IR camera are shown in Figure 11 and Table 4.

All temperature results are presented as the excess of ambient temperature. The temperature of the LED is much lower due to the significant amount of optical energy emitted. The main achieved results confirm the high optical energy efficiency of the tested LED, reaching η ≈ 30%.

**Figure 12:**
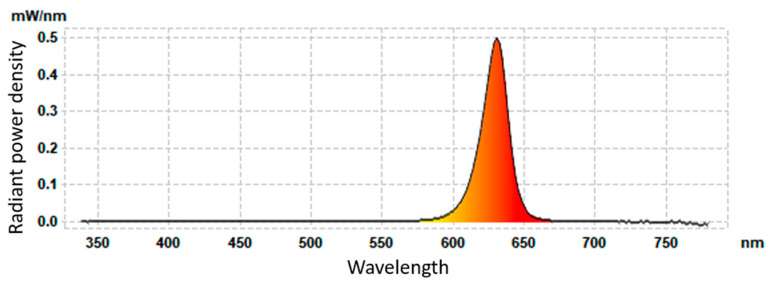
Power density of emitted LED radiation vs. wavelength for *I* = 20 mA.

Using the integrating sphere, it was possible to measure both the spectrum and the power density of the emitted radiation within the 350–750 nm wavelength range [1]. The spectrum of emitted LED radiation at a current of *I* = 20 mA is shown in Figure 12. It confirms the narrow band of the radiation and the high optical power effectiveness of the tested LED. The optical power measured with the integrating sphere was *P_opt_* = 11.3 mW and agrees with the thermographic measurement very well. The optical efficiency of the tested LED was high and estimated at η≈30%. This result agrees with the manufacturer’s data.

**Figure 13:**
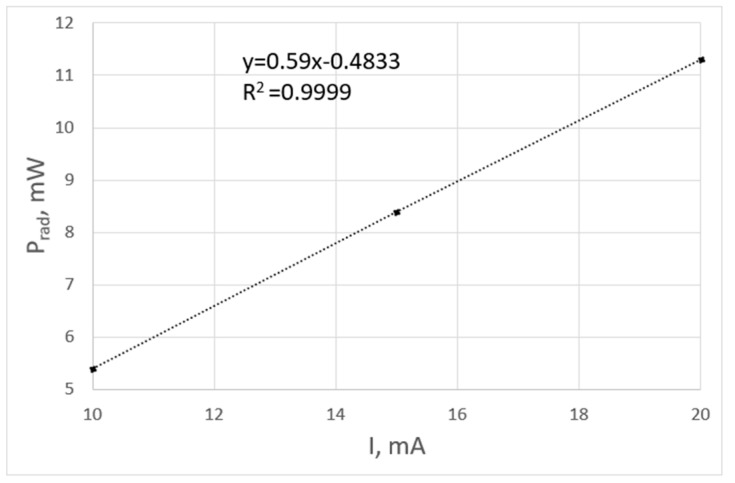
Optical power of a diode as a function of the current.

The measurements carried out in the integrating sphere for different currents flowing through the diode confirm the linear relationship between the optical power generated and the current of the diode—Figure 13.

### 3.3. Uncertainty Analysis

The measurement uncertainty was calculated according to the specifications given in the literature [14,15,16,17]. Optical power (19) depends on the temperature and both the electrical power supplied to the diode and the resistor and the thermal power dissipated in them. It was assumed that the measurement uncertainty of radiant power depends on the uncertainty of measurements of *T_R_* and *T_D_* and the uncertainty of power dissipated in the connecting wires—Equation (20). The uncertainty of electrical power is negligible because this power was measured using professional electrical quantity meters with a high accuracy and a high resolution.

In order to obtain reliable results from thermographic measurements, it is necessary to determine the maximum permissible range of variability in the measured quantity with a given probability, called the confidence level. This value refers to the expanded uncertainty, denoted as *U*. Expanded uncertainty provides the range of potential variation in measurement results due to random fluctuations in the measured data and systematic errors of the apparatus used. It uses a coverage factor to increase uncertainty as the number of measurements decreases.

The expanded uncertainty of the optical power measurement can be expressed as:

(20) $$U_{P_{opt}}=k_{p}u_{c}(P_{opt}=f(T_{D},T_{R},P_{12D},P_{12R}))$$

where *k_p_* is the coverage factor depending on the number of measurements performed and *u_c_* is the combined uncertainty.

The combined uncertainty can be expressed as:

(21) $$u_{c}(P_{opt})=\sqrt{{\left(\frac{\partial P_{opt}}{\partial T_{D}}u_{c}(T_{D})\right)}^{2}+{\left(\frac{\partial P_{opt}}{\partial T_{R}}u_{c}(T_{R})\right)}^{2}+{\left(\frac{\partial P_{opt}}{\partial P_{12D}}u_{c}(P_{12D})\right)}^{2}+{\left(\frac{\partial P_{opt}}{\partial P_{12R}}u_{c}(P_{12R})\right)}^{2}}$$

The combined uncertainty consists of A- and B-type uncertainties, *u_A_* and *u_B_*, relating to the stochastic nature of the measurement and the maximum absolute error of the measurement devices.

(22) $$u_{c}=\sqrt{{u_{A}}^{2}+{u_{B}}^{2}}$$

Both uncertainties *u_A_* and *u_B_* were calculated independently for both temperature values of the diode *T_D_* and the resistor *T_R_*. The entire measurement process consisted of six sessions in which six measurements were taken in order to average the results of the measured quantities. The entire measurement lasted for several hours at a variable ambient temperature. A variable ambient temperature is one of the main sources of increasing uncertainty in temperature measurements with bolometric cameras. Averaging the measurement results allowed us to reduce the type A uncertainty of the temperature measurement significantly.

(23) $$u_{A}(x)=\sqrt{\frac{\sum_{i=1}^{N}{\left(x_{i}-\bar{x}\right)}^{2}}{N(N-1)}}$$

where *x* is the measured value (temperature of the diode and resistor and power of the connecting wires) for each measurement and $\bar{x}$ is its average value. The symbol *x* refers to the temperature of the diode, *T_D_*, and the resistor, *T_R_*, and the power, *P*_12*R*_ and *P*_12*D*_, and *N* = 6 means the number of measurements in each session.

Note that all temperature values presented in this work refer to the excess in temperature over the ambient temperature. Type A uncertainty results are shown in Table 5.

The systematic error component of uncertainty *u_B_*(*T*)—type B uncertainty—is determined by the maximum absolute error Δ*T_max_* of a single measurement from the thermal imaging camera. It was assumed that the source of this measurement uncertainty is the IR camera itself [16,17]. The accuracy of the thermal camera used in this work is Δ*T_max_* = 2% of the measured value.

(24) $$u_{B}(T)=\frac{∆T_{max}}{\sqrt{3}}=\frac{0.02T}{\sqrt{3}}$$

For the power dissipated in the connecting cables, the uncertainty *u_B_* is scaled by the factor *kS/*Δ*x*, where *k* is the thermal conductivity of the cable, *S = πr*^2^ is the cross-sectional area, and Δ*x* is the length of the cable for which the gradient is calculated.

(25) $$u_{B}(P)=\frac{kS}{\Delta x}\frac{∆T_{max}}{\sqrt{3}}=\frac{kS}{∆x}\frac{0.02T}{\sqrt{3}}$$

where *k* = 200 W/(m·K), Δ*x* = 1.26 cm, *S = πr*^2^, and *r* = 0.1 mm.

According to uncertainty standards [17,18], if the uncertainty of *u_B_* dominates over *u_A_*, then the random variable has a uniform probability distribution and the value of the coefficient *k_p_* (20) is equal to $\sqrt{3}$ for a 100% confidence level.

Based on (19) and (21), the combined uncertainty of the optical power measurement *u_c_* is estimated as:


(26) $$u_{c}(P_{opt)}=\sqrt{{\left(\frac{T_{D}(P_{elR}-P_{12R})}{{T_{R}}^{2}}u_{c}(T_{R})\right)}^{2}+{\left(\frac{\left(P_{elR}-P_{12R}\right)}{T_{R}}u_{c}(T_{D})\right)}^{2}+u_{c}^{2}(P_{12D})+{\left(\frac{T_{D}}{T_{R}}u_{c}(P_{12R})\right)}^{2}}$$

Using actual measurement values, *U* = 1.17 mW was estimated, so the final result of estimating the LED optical power is *P_opt_* = 11.08 ± 1.17 mW for a thermal camera with a 2% accuracy. When this accuracy is lowered to 1%, the uncertainty *U* = 0.92 mW. In order to further reduce the uncertainty, it is necessary to increase the number of measurements that are averaged.

## 4. Conclusions

This article presents a simple engineering method for evaluating the optical power of LEDs by measuring the temperature using infrared thermography. The presented results seem promising. In addition, during this research, a compact thermal model in the form of a network of thermal resistances was constructed. Advanced 3D-FEM thermal modelling was performed in the ANSYS environment to compare the results with the measurements. In order to verify the obtained results, the optical power of the diode was measured using an integrating sphere. The results were very close to each other. In order to show the practical potential of the proposed new thermographic method of measuring the optical power of LEDs, an uncertainty analysis was carried out. This analysis shows how the accuracy of such measurements can be improved. First, the accuracy of the IR camera must be high, at least 1%. The temperature then needs to be averaged over both time and space. It is recommended to choose an area of interest containing at least a dozen pixels of the thermal image to average the temperature.

The proposed method of measuring the optical power of LEDs is an engineering alternative to using an integration sphere and spectrometer that can only be used in the laboratory, as it cannot be used remotely, but, on the other hand, it is very accurate. It needs expensive equipment, requires calibration, and cannot be used while diodes are operating in a system.

## Figures and Tables

**Table 1:** Parameters of the compact thermal model of the LED.

Parameter	Value	Description
*k_epoxy_*	2 W/(m·K)	Thermal conductivity of epoxy resin
*k_Cu_*	300 W/(m·K)	Thermal conductivity of metal contacts containing mainly copper (wire)
*S_d_*	4.92 × 4.92 × 10−^6^ m^2^	Diode top and bottom surface
*S*_1*e*_	3 × 3 × 10^−6^ m^2^	Epoxy resin replaced cross-section surface
*S*_2*e*_	4.92 × 2.7 × 10^−6^ m^2^	Left and right diode surface
*S*_3*e*_	4.92 × 3 × 10^−6^ m^2^	Side surfaces of the diode
*r*	300 × 10^−6^ m	Connecting wire radius
*d*_1_	1.5 × 10^−3^ m	Half the thickness of the epoxy layer
*d*_2_	2 × 10^−3^ m	The length of the inner connector to the outer conductor
*d_Cu_*	2.5 × 10^−3^ m	The length of the copper pad on which the diode is placed
*h*_1_	10.61 W/m^2^K	Heat transfer coefficient for the top surface
*h*_2_	5.96 W/m^2^K	Heat transfer coefficient for the bottom surface
*h*_3_	13.33 W/m^2^K	Heat transfer coefficient for the side’s surfaces
*h*	12.09 W/m^2^K	Heat transfer coefficient for the connecting wire

**Table 2:** Temperature in all nodes obtained by the R_th_ network compact model.

Node No	1	2	3	4	5	6
ΔT, °C	7.07	6.93	6.99	7.07	7.07	6.98

**Table 3:** Temperature obtained by the 3D FEM thermal model.

Measuring Point	Heat Source	Top Surface	Bottom Surface	Electrical Contact 1	Electrical Contact 2	Side Surfaces
ΔT°C	7.46	6.95	7.02	7.26	7.24	7.11

**Table 4:** Measurement results of temperature *T_D_* and *T_R_* and optical power *P_opt_* for a supplying current of *I* = 20 mA for six measurement sessions lasting a few hours. The last row presents mean values.

P_elD_ mW	P_elR_ mW	T_amb_ °C	T_d_ °C	T_r_ °C	ΔT_d_ = T_D_ °C	ΔT_r_ = T_R_ °C	P_12D_ mW	P_12R_ mW	P_rad_ mW	η %
38.7	87.3	26.3	33.8	47.7	7.05	20.95	1.5	11	11.55	29.80
38.7	87.3	26.7	33.77	47.6	7.02	20.85	1.6	9	10.77	27.79
38.8	87.6	27.3	33.73	47.72	6.98	20.97	1.5	9	11.15	28.77
38.7	87.3	26.9	33.67	47.70	6.92	20.95	1	9	11.88	30.66
38.7	87.2	26.8	33.47	47.52	6.72	20.77	3	10	10.72	27.66
38.8	87.5	26.5	33.62	47.7	6.87	20.95	3	10	10.39	26.82
**38.8**	**87.4**	**26.75**	**33.68**	**47.66**	**6.93**	**20.91**	**1.93**	**9.67**	**11.08**	**28.58**

**Table 5:** Uncertainties *u_A_*, *u_B_*, and *u_c_* for measurements of temperature, *T_D_* and *T_R_*, as well as for power dissipated in the connecting wires, *P*_12*D*_ and *P*_12*R*_.

Parameter	Mean Value	*u_A_*	*u_B_*	*u_c_*
*T_D_* (°C)	6.93	0.05	0.08	0.09
*T_R_* (°C)	20.91	0.03	0.24	0.24
*P*_12*D*_ (mW)	1.93	0.35	0.00	0.35
*P*_12*R*_ (mW)	9.67	0.33	0.00	0.33

## Data Availability

Dataset available on request from the authors.
